# Long-Term Follow-Up of Retromuscular Incisional Hernia Repairs: Recurrence and Quality of Life

**DOI:** 10.1007/s00268-017-4268-0

**Published:** 2017-10-10

**Authors:** Peder Rogmark, Sam Smedberg, Agneta Montgomery

**Affiliations:** 1 Department of Surgery, Skåne University Hospital, 205 02 Malmö, Sweden; 2 Institution for Clinical Sciences Malmö, Lund University, Lund, Sweden; 3 Department of Surgery, Helsingborg Hospital, Helsingborg, Sweden

## Abstract

**Purpose:**

Incisional hernia repair (IHR) with a mesh is necessary to achieve low recurrence rates and pain relief. In the short term, quality of life (QoL) is restored by IHR. Two centers pioneered the IHR in Sweden with the highly standardized Rives–Stoppa technique using a retromuscular mesh. We assessed long-term follow-up of recurrence rate and QoL.

**Methods:**

Medical records were searched for IHRs performed from 1998 to 2006 and included living patients with midline repairs. Questionnaires about physical status, complaints, and QoL (SF-36) were mailed, offering a clinical examination. Assessment of medical records of later surgery was performed in 2015.

**Results:**

Three hundred and one patients with midline incisional repairs were identified, and 217 accepted participation. Of these, 103 attended a clinical examination. Follow-up was 7 years until examination and 11 years to reassessment of medical records. In 26%, recurrent hernias were repaired. Postoperative complications were 26% Clavien–Dindo grade I–II and 1% grade III–IV. Mesh infections occurred in 1.4% without mesh removals, and 4% were reoperated because of complications. Overall recurrence rate was 8.1% and two-third of which were diagnosed at clinical examination. Recurrence after primary and recurrent hernia repair was 7.1 and 10.9%, respectively. Of all patients, 80% were satisfied; dissatisfaction was primarily caused by recurrence and chronic pain. SF-36 scores were 0.2 SD lower than the norm in all subscales, similar to those with 1–2 chronic conditions.

**Conclusions:**

Midline retromuscular mesh IHR has a low long-term recurrence rate even after recurrent repair. Patient satisfaction was high although QoL was reduced.

## Introduction

The prevalence of developing an incisional hernia is estimated to be 13% 2 years after a midline incision according to a recent meta-analysis [1]. Though not every hernia is symptomatic and requires surgery, the repair of incisional hernias is common, and almost 80% will eventually need a repair [2]. The Rives–Stoppa (RS) surgical technique of retromuscular mesh implantation was introduced in Sweden in 1995. Two neighboring hospitals pioneered the development of mesh incisional hernia repair (IHR) in Sweden. Since 1998, it has been the procedure of choice for midline IHR using a highly standardized technique. With growing experience, these hospitals received many referred patients with complex abdominal wall hernias.

The retromuscular mesh implant utilizes the anatomical space dorsal to the abdominal rectus muscles which is virtually free from nerves and blood vessels, and intercostal nerves and vessels are at the lateral border of the rectus muscle. The epigastric vessels run along the back of the rectus muscle from below and above, are easy to identify, and are sparse. The RS repair provides sufficient mesh overlap at the lateral borders of the hernia defect in most cases.

Recurrence after the RS technique varies between 5 and 24% and occurs more frequently after a former repair [3–5]. Chronic pain levels, reported in two studies, varied between 20 and 27%. A corresponding fraction of satisfied patients (77–89%) was reported [3, 5].

We conducted an RCT comparing IPOM to RS on quality of life (QoL) before surgery and 8 weeks and 1 year after surgery. Low QoL before surgery was resolved after 8 weeks and remained comparable to the norm in all domains at 1 year. Long-term QoL after RS repair is scarcely reported. Langbach et al. [6] used the SF-36 instrument to evaluate QoL, comparing IPOM to RS and a non-operated group. After 50-month follow-up, no difference in QoL was found between IPOM and RS. Operated patients reported better physical function compared to controls, and QoL was significantly reduced in patients suffering from a recurrence.

This study aimed to investigate the long-term QoL as measured by the SF-36, recurrence rate and remaining complaints after midline IHR using a standardized RS procedure with a polypropylene mesh.

## Patients and methods

The patient databases in two hospitals were searched for any patient discharged between 1998 and 2006 with a surgical procedure code indicating a planned IHR. Approval by the Regional Ethics Review Board in Lund was obtained (340/2008).

All living patients received by mail information about the study, an informed consent form, a QoL questionnaire, a specific survey on complaints, and an invitation to a free clinical examination. Patients who declined participation could specify *No complaints what so ever from my abdominal wall*. Non-responders received two reminders.

### Operative technique

In the RS technique, the aponeurosis of the rectus muscle was opened medially on both sides and at least 5 cm above and below the hernia defect. After mobilizing the dorsal sheath and closing the midline to protect the abdominal contents, a heavyweight monofilament polypropylene mesh was placed to cover the retro-rectus space. The dominant meshes used were Surgipro™ (Covidien), Atrium Mesh, and Prolene™ (Ethicon). At least 5 cm overlap was desired. The mesh was fixed with a running non-resorbable suture to the midline. No lateral sutures or transfixing sutures were used. The borders of the anterior aponeurosis were approximated for closure, and if tension was too high, the borders were sutured to the mesh, leaving a small part of the mesh exposed to the subcutaneous tissue. The wound was closed with subcutaneous sutures and superficial sutures or clips. Subcutaneous drains were used when deemed necessary.

### Medical records

Medical records were searched for operative data (age, sex, ASA classification, recurrent hernia, hernia location, operation time, operating surgeon, elective or emergency setting, total hospital stay, and reoperations). Complications were classified according to the Clavien–Dindo scale [7]. A postoperative diagnosis of recurrence at abdominal wall examination or a reoperation for recurrence was noted.

In 2015, the medical records were reviewed for any later recurrence or abdominal surgery.

### Questionnaires

The SF-36 was used as the health-related QoL instrument (licensed by the HRQOL-group at Gothenburg University). It is reported as norm-based scores with mean (Mn) 50 and standard deviation (SD) 10. We used the Swedish population as norm base [8].

A “patient friendly” easy to use abdominal wall questionnaire developed for this trial assessed the weight and height and whether the respondent was fully recovered (yes/no), the frequency of abdominal wall complaints (rarely/monthly/weekly/daily), and whether analgesics were needed for these complaints (yes/no). Complaints were recorded for three elements, physical pain, movement limitation, and fatigue, using a 100-mm visual analogue scale (VAS). Each was graded from *No complaint at all* to either *Unbearable* (pain)*, Cannot move* (movement limitation), or *Totally exhausted* (fatigue), respectively. Types of complaints were categorized as stiffness, skin problems, cosmesis, or clothing problems based on yes/no responses.

Patient satisfaction and whether the respondent would proceed with the operation if allowed to go back, given their current experience, were measured using 4-point scales. Allowed responses were *definitely positive, positive, negative,* or *definitely negative*.

### Clinical examination

Long-term follow-up with a clinical examination was performed from 2010 to 2012. If any recurrent hernia was detected or suspected at the examination, and if it was a clinical problem and the patient was interested in a further surgical intervention, a CT scan was offered.

### Statistics

Statistic hypothesis tests were performed using the *χ* ^2^ or Fisher exact test for nominal variables and the Student *t* test or Mann–Whitney test for continuous variables, depending on the distribution. Ordinal data were analyzed using the Kendall *τ* rank correlation. Skewed distributions are reported using the Mn and SD as well as the Md and interquartile range (IQR).

### Literature search

A search of comparable published work focused on similar retromuscular techniques, proportions of primary and recurrent hernias, recurrence rates, mesh explantation rates, QoL data using both a norm-based SF-36 and patient satisfaction assessment, and long-term follow-up.

## Results

A total of 596 patients were identified with any abdominal wall repair. Medical records revealed that 31 did not present a midline incisional hernia, and 70 did not receive a retromuscular mesh via the RS technique. They were therefore excluded. Medical records of 194 deceased patients were not available because they had been removed from the computerized systems.

**Fig. 1:**
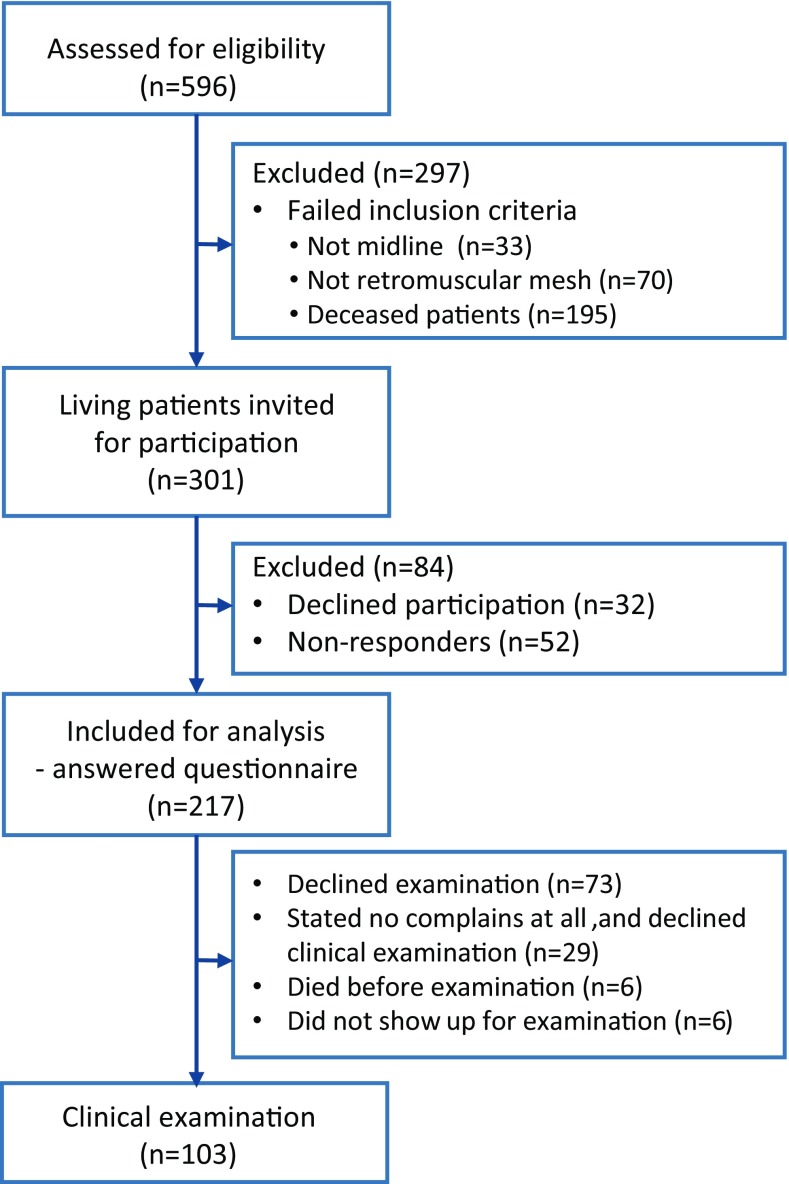
Study flowchart

The 301 patients still living were included and invited; 32 declined participation; and 52 did not respond after two reminders and were considered lost to follow up. The remaining 217 patients were included for analysis. Twenty-nine patients (13%) reported no complaints at all and did not complete the questionnaires. Twelve of these were referral patients living in other regions in Sweden. The invitation to a clinical examination was agreed to by 117, and 103 fulfilled the agreement. Prior to examination, 6 patients died. Patient cohort selection is summarized in Fig. 1.

The Mn (SD) follow-up was 87 (31) months, and the Md (IQR) follow-up was 84 (62–112) months until clinical examination, and an updating of medical record review was performed 50 months later, making the Mn (SD) follow-up 137 (31) months.

### Medical records

At the time of surgery, Mn (SD) patient age was 61 (13) years and 56% were women. Patients were classified ASA-I in 19%, ASA-II in 71%, and ASA-III in 10%. In 26%, a recurrent incisional hernia was repaired. A total midline incision was repaired in 34%, an upper midline incision in 25%, an umbilical incision in 4%, and a lower midline in 29%. Mean (SD) operating time was 144 (69) minutes around a Md (IQR) of 132 min (98–174 min). Mean (SD) hospital stay was 3.9 (3.54) days around a Md (IQR) of 3 days (2–5 days).

Postoperative complications were recorded in 27% of the patients; 12% were Clavien–Dindo grade I, 14% grade II, and 1% grade IIIb. One patient suffered severe postoperative respiratory insufficiency and received a tracheostomy, spending 29 days in the intensive care unit. This was a Clavien–Dindo grade IV complication.

A total of 9 (4%) patients were reoperated. Three were reoperated within 30 days, 1 each for arterial bleeding from the rectus muscles, hematoma, and umbilical skin necrosis. An additional 6 were reoperated within 90 days, 3 (1.4%) for deep wound (mesh) infections, and 1 each for a small bowel obstruction in a parastomal hernia, migrant thrombophlebitis, and severe neuropathic pain (exploratory). The deep infections were treated with antibiotics, dressing changes, and negative pressure wound therapy. No meshes were explanted.

Five surgeons performed 79% of the operations and assisted in a further 20% of them. Three (1.4%) emergency operations were performed without an expert surgeon.

### Questionnaires

**Fig. 2:**
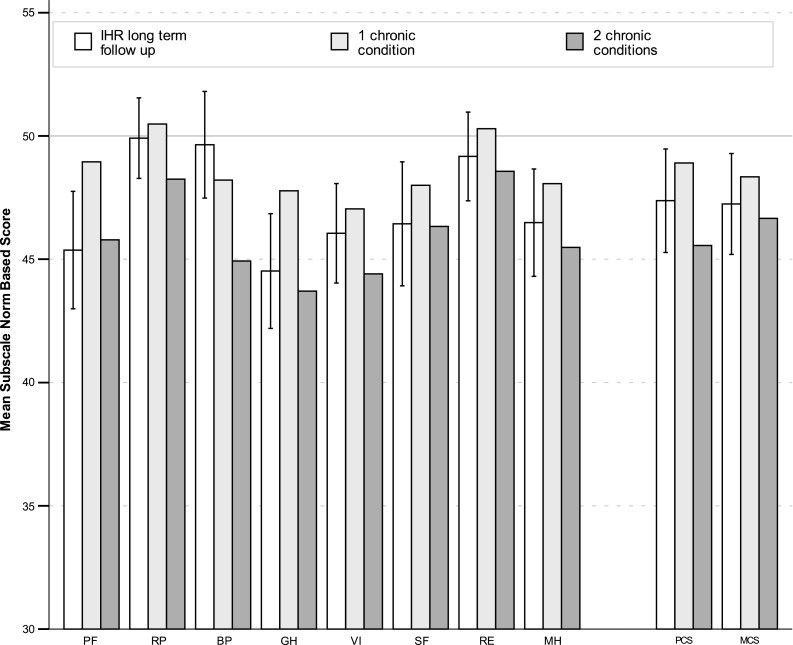
SF-36 scores. SF-36 subscales and composite scores with 95% confidence intervals. Scores from norm group patients with 1 and 2 registered chronic conditions [18] included for comparison. Norm is 50 SD 10. *PF* physical function, *RP* role physical, *BP* bodily pain, *GH* general health, *VT* vitality, *SF* social function, *RE* role emotional, *MH* mental health, *PCS*/*MCS* physical/mental composite score

Results of the SF-36 questionnaire are reported in Table 1 and Fig. 2. Both composite scores and all subscales, except role physical, bodily pain, and role emotional, were reduced from the norm level by at least 2 points. Cohen’s *d* [9], the standardized effect size often used in psychometric instruments, empirically defines a small clinical significant difference as 0.2 SD and a moderate 0.5 SD, which in SF-36 scales (with SD 10) correspond to 2 and 5 points, respectively.

**Table 1:** SF-36 scores

				Number of chronic conditions (Taft et al. [16])^a^			
Subscale	Mean	SD	SEM	None	1	2	3+
PF	45.4	12.2	1.20	53.0	49.0	45.8	37.8
RP	49.9	8.4	0.82	53.0	50.5	48.2	42.9
BP	49.6	11.1	1.09	52.5	48.2	44.9	40.1
GH	44.5	11.9	1.17	52.6	47.8	43.7	37.0
VI	46.0	10.3	1.02	49.9	47.0	44.4	39.8
SF	46.4	12.9	1.27	51.0	48.0	46.3	39.7
RE	49.2	9.2	0.91	52.2	50.3	48.6	45.1
MH	46.5	11.1	1.10	50.1	48.1	45.5	42.0
PCS	47.4	10.7	1.06	53.3^b^	48.9^b^	45.6^b^	38.4^b^
MCS	47.2	10.5	1.03	49.9^b^	48.3^b^	46.7^b^	43.7^b^

*PF* physical function, *RP* role physical, *BP* bodily pain, *GH* general health, *VT* vitality, *SF* social function, *RE* role emotional, *MH* mental health, *PCS/MCS* physical/mental composite score

^a^Scores were *z*- and *t*-transformed for norm-based values

^b^PCS and MCS calculated from mean subscale scores

In the specific questionnaire, abdominal wall complaints were reported as *rarely* by 72%, *monthly* by 9.4%, *weekly* by 4.3%, and *daily* by 14.5% of the patients. Mean (SD) VAS pain score was 13 (22) mm around Md (IQR) of 3 mm (0–15 mm), and 87% scored 30 or less. Mean (SD) perceived *fatigue* and *movement limitation* VAS scores were 10 (2) and 11 (2) mm, respectively, and 89 and 86%, respectively, scored less than 30. Approximately 76% of the patients reported recovered abdominal walls. A total of 9.8% continued to use analgesics on a daily basis because of abdominal wall symptoms. About 64% were *definitely satisfied*, 16% *satisfied*, 12% *not satisfied*, and 8% *definitely not satisfied* with the hernia repair. Half of those who were *definitely not satisfied* suffered a recurrence, and all had chronic or intermittent pain in the operated area. In all, 80% were satisfied.

### Clinical examination at long-term follow-up

Overall recurrence rate was 8.1% (*n* = 17); 3.3% (*n* = 6) were diagnosed via medical records; and 4.8% (*n* = 11) were found at the clinical examination. One patient had a recurrent hernia and a second repair. No patient had records of recurrence or of abdominal surgery at the review in 2015. Recurrences were identified in 7.1% after primary IHR and in 10.9% after recurrent IHR (*p* < 0.366).

### Patients not clinically examined

Compared to examined patients, those who were not clinically examined did not differ in terms of surgical unit, age, BMI, number of hernia defects repaired, operative time, Clavien–Dindo grading, presence of chronic pain, or in the physical and mental composite scores in the SF-36 (*p* > 0.05). The VAS measures were imputed as 0 for the 29 patients who claimed no complaints. Only in the VAS pain score was a difference found in Mn 4 mm for the whole cohort of examined patients versus 16 mm those with no complaints (*p* < 0.001).

## Discussion

Follow-up after retromuscular mesh IHR in this study is one of the longest reported, spanning more than 11 years and focusing on recurrence and QoL. In this study, the procedures were highly standardized and heavyweight polypropylene meshes were routinely used. All planned operations were performed by expert hernia surgeons.

In patient medical records, only a few recurrences were noted, likely the result of local symptoms reported by the patients. A recurrence rate of 8.1% after 11 years compares favorably to several other long-term studies (see Table 2). Two studies with a similar proportion of operated recurrent hernia patients reported very different recurrence rates: Iqbal et al. [5] reported 5% after 6 years and Hawn et al. [4] reported 34% after 5 years. Several other authors have published recurrence rates around 30% [3, 10, 11]. Most recurrence rates reported after IHR were higher during the first 2–3 years [6, 10–12], and the rate decreased thereafter but never reached 0.

**Table 2:** Long-term follow-up on retromuscular mesh incisional hernia repair

Study	Trial type	*N*	Time period	Recurrent hernia op. (%)	Follow-up (months)	Explanted mesh (%)	Recurrence rate (%)	Patients satisfied (%)
Burger et al. [3]	RCT	84	1992–1998	12	81	0	32	77
Yaghoobi Notash et al. [18]	R	86	1993–2003	28	68	1.1	6	–
Ballem et al. [10]	R	106	1996–2001	–	60	–	28	–
Iqbal et al. [5]	R	254	1990–2003	35	70	2.0	5	89
Paajanen et al. [6]	R	84	1997–2000	19	36	0	5	87
Hawn et al. [4]	REG	916	1997–2002	26	60	1.7	34	–
Kokotovic et al. [13]	REG	1119	2007–2010	18	59	1.6	13	–
Langbach et al. [12]	R	73	2000–2010	18	52	–	23	48
Singhal et al. [11]	R	127	2001–2006	30	48	–	28	–
Rogmark (*Current study*)	R	217	1998–2006	26	137	0	8	80

Values are rounded to integers

*RCT* randomized controlled trial, *R* retrospective study, *REG* register study

The study from the national Danish Hernia Register [13] reported 13% reoperation rate. Registers that do not include a clinical examination fail to detect the “true” recurrence rate. Using only reoperation as a proxy for recurrence rate detects only 20–25% of the cases [14]. In our trial, clinical examination revealed almost twice as many recurrences when clinical examination was added. Most were without local symptoms and thus without surgical indication. This corresponds to the findings by Kald et al. [15], who showed that reoperation as a surrogate for recurrence in inguinal hernias underestimates the clinical recurrence rate by 38%.

A small pore mesh was the standard at the time to secure for not having a problem of bulging, especially if bridging was necessary. Salvage of all meshes, even when deep mesh infections occurred, was possible in all patients in this study. This is somehow unusual, but has also been reported by Burger et al. [3] and Paajanen et al. [16] (Table 2). This might be due to the fact of using techniques where meshes are only minimally exposed to subcutaneous tissue and to the use of modern wound care, including vacuum-assisted techniques.

This study has some obvious limitations. It is a retrospective investigation in a field where neither detailed descriptions of the hernia nor follow-up evaluations were standardized. Many patients were lost, either dead or ailing from other diseases, or had moved beyond our reach, making some data too weak to infer conclusions. In all, 35% (103/296) of the living patients were clinically examined compared to 50% (71/142) by Burger et al. [3].

Postoperative complications are correlated to recurrence [5], and the most common complication after open IHR is wound infection [10, 17]. The necessity to explant a mesh in an infected wound is dependent on the mesh material. In this study, no meshes were removed because a (heavyweight) monofilament polypropylene mesh was used. Mesh explantation rates seem to be 1–2% due to infection, but this is also dependent on the type of mesh used (see Table 2).

Patient satisfaction was positive in 80% after 87 months, slightly lower than the 92% found after 12 months in our RCT PROLOVE [12] on laparoscopic versus open IHR. Iqbal et al. [5] noted 89% satisfied patients, but Langbach et al. [6] only 48%. Two important differences are found between these studies. First is the recurrence rate (5 vs. 28%), and second is the proportion of operated recurrent hernias (35 vs. 18%). Recurrent incisional hernias are usually more complex and have higher recurrence rates than primary incisional hernias [4]. However, in this study we did not find a difference in recurrence rates between primary and recurrent incisional hernias, coinciding with the findings by Singhal et al. [11]. For obvious reasons, satisfaction decreases when the patient suffers from chronic pain or when a recurrence occurs [6].

After 87 months, QoL was lower in 5 out of 8 dimensions in the SF-36 also reflected in both composite scores. The pattern is roughly the same 1 year after surgery [12], but with generally lower scores. Taft et al. [18] assessed the performance of the SF-36v2 in Sweden and reported data on several subgroups. The SF-36v2 has higher resolution in some subscales, but scores are comparable between versions 1 and 2 [19]. Our results compare well to the subgroup with two chronic conditions. It is reasonable that comorbidities other than the repaired incisional hernia (or the preceding surgical indication) affect QoL, considering that the mean age at follow-up was 69 years.

## Conclusions

The retromuscular RS repair of midline incisional hernias has a low and acceptable recurrence rate in the long-term perspective and is equally efficient when treating recurrent incisional hernias. About one-third of diagnosed recurrences were reoperated, all without mesh removal. Patient satisfaction is high, provided chronic conditions, such as recurrence or pain, do not occur. Quality of life is reduced to a level similar to that experienced by patients with two chronic conditions.
